# Politics–evidence conflict in national health policy making in Africa: a scoping review

**DOI:** 10.1186/s12961-024-01129-3

**Published:** 2024-04-15

**Authors:** Edward W. Ansah, Samuel Maneen, Anastasia Ephraim, Janet E. Y. Ocloo, Mabel N. Barnes, Nkosi N. Botha

**Affiliations:** Department of Health, Physical Education and Recreation (HPER), University of Cape Coast, Cape Coast, Ghana

**Keywords:** Health policy, Health politics, Policy-making, Evidence-based policy, Public health, Public policy, Africa

## Abstract

**Background:**

Generally, public health policy-making is hardly a linear process and is characterized by interactions among politicians, institutions, researchers, technocrats and practitioners from diverse fields, as well as brokers, interest groups, financiers and a gamut of other actors. Meanwhile, most public health policies and systems in Africa appear to be built loosely on technical and scientific evidence, but with high political systems and ideologies. While studies on national health policies in Africa are growing, there seems to be inadequate evidence mapping on common themes and concepts across existing literature.

**Purpose:**

The study seeks to explore the extent and type of evidence that exist on the conflict between politics and scientific evidence in the national health policy-making processes in Africa.

**Methods:**

A thorough literature search was done in PubMed, Cochrane Library, ScienceDirect, Dimensions, Taylor and Francis, Chicago Journals, Emerald Insight, JSTOR and Google Scholar. In total, 43 peer-reviewed articles were eligible and used for this review.

**Result:**

We found that the conflicts to evidence usage in policy-making include competing interests and lack of commitment; global policy goals, interest/influence, power imbalance and funding, morals; and evidence-based approaches, self-sufficiency, collaboration among actors, policy priorities and existing structures. Barriers to the health policy process include fragmentation among actors, poor advocacy, lack of clarity on the agenda, inadequate evidence, inadequate consultation and corruption. The impact of the politics–evidence conflict includes policy agenda abrogation, suboptimal policy development success and policy implementation inadequacies.

**Conclusions:**

We report that political interests in most cases influence policy-makers and other stakeholders to prioritize financial gains over the use of research evidence to policy goals and targets. This situation has the tendency for inadequate health policies with poor implementation gaps. Addressing these issues requires incorporating relevant evidence into health policies, making strong leadership, effective governance and a commitment to public health.

## Introduction

The state of human health varies globally, such that long-term human security is endangered at multiple levels [1, 2]. Current and future efforts aimed at containing and improving population health and well-being are expressed in many national health policies [3]. A national health policy is a planned course of action carried out by a country or state to attain defined healthcare goals [4–6]. Perhaps, public policy-making and its processes are at the instance of the political leaders [1]. Generally, public policy-making is hardly a linear process, characterized by interactions between and among politicians, institutions, researchers, technocrats and practitioners from varied fields, as well as brokers, interest groups and a gamut of other actors [5]. Right from its inception through to review, the policy-making process can be burdened with serious rifts, especially among the principal actors such as politicians, researchers and technocrats [7] which could place limitations on the use of evidence to inform the policy.

There are several frameworks for policy analysis, but the health policy triangle (HPT) by Walt and Gilson [8, 9] gained popularity. The HPT consists of four main constructs, including the policy context, policy content, policy process and the policy actors. The policy context refers to the socioeconomic, political, cultural and other environmental variables that necessitate the policy [8, 9]. The policy content refers to the core policy objectives, regulations and legislations that underpinned the policy. The policy process refers to how the policy evolves, from conception, formulation, negotiation, communication and implementation to evaluation. Moreover, policy actors refer to significant individuals, groups and institutions that influence the policy process [8, 9]. Thus, critically following the tenets of this framework may lead to formulation and implementation of health promoting policies that help lesson the burden of ill health on Africa. Meanwhile, a robust and evidence-based health policy is most likely to guarantee equitable and optimum human health [10].

Furthermore, it is believed that health policies in Africa are driven largely by political rather than technical interests backed by robust evidence [3]. However, factors such as funding, scientific evidence, interest and activities of lobbyists, and political interest/commitment influence national health policies [3, 11]. The policy-making process, either driven by need, evidence based or political interest, determines the core policy objectives and how they are attained. Thus, poorly crafted and implemented health policies in Africa, for instance, challenge access to quality health because of poor funding, corruption, equity and quality gaps, making such policies ill-prepared for national emergencies [1, 7, 10]. Meanwhile, universal health coverage by WHO, re-echoed in the Sustainable Development Goals (SDGs), remains a dream for the majority of countries in Africa [1, 12]. Therefore, this conflict between the use of political goals and objectives over research evidence in public health policy-making needs exploration.

Several studies have revealed that public health policies and systems appear to be built loosely on technical and scientific evidence, but with high political systems and ideologies [13–16]. For instance, in most African countries such as Ghana, Kenya, Nigeria, South Africa, Tunisia, and Zimbabwe, the management of the novel SARS-CoV-2 pandemic and the public health response have been heavily politicized [16–18]. Regardless of the seeming increase in research evidence on the central role of political leadership in defining national public health policies in Africa, there seems to be inadequate empirical accounts of the common themes or concepts in the existing literature regarding the politics–evidence conflict in the public health policy-making process [18, 19]. For instance, though evidence exists of the advances in knowledge about evidence-led health policies [19], research on the politics–evidence relationship remains largely unclear. Furthermore, while studies on health systems strengthening gave prominence to the role of technical knowledge in public health policy effectiveness, evidence on how politics shapes health systems is unclear [20, 21]. However, such health development in Africa is largely driven by politics [16–18]. Clearly, there is the need to establish the common themes and concepts that cut across the existing literature on the subject under discussion. Therefore, to fill this research gap, this review scoping explores the extent of evidence in relation to politics and scientific evidence utilization in the national health policy-making process in Africa.

## Materials and methods

We utilized only peer-reviewed articles to examine the relationship between politics and scientific evidence in the national health policy-making processes in Africa. We utilized the approach of Tricco et al. [22] in probing, synthesizing and analysing appropriate peer-reviewed articles. The approach includes (i) outlining and developing the purpose of the review, (ii) outlining and critically examining the review questions, (iii) identifying and scrutinizing article search terms, (iv) identifying and exploring related databases and downloading useful articles, (v) screening the data, (vi) summarizing the data and reconciling the results, and (vii) consulting [22]. Therefore, two research questions informed the review: (1) what is the nature of politics–evidence conflict in national health policy-making in Africa? and (2) what are the challenges with politics–evidence conflict in national health policy-making in Africa?

**Fig. 1:**
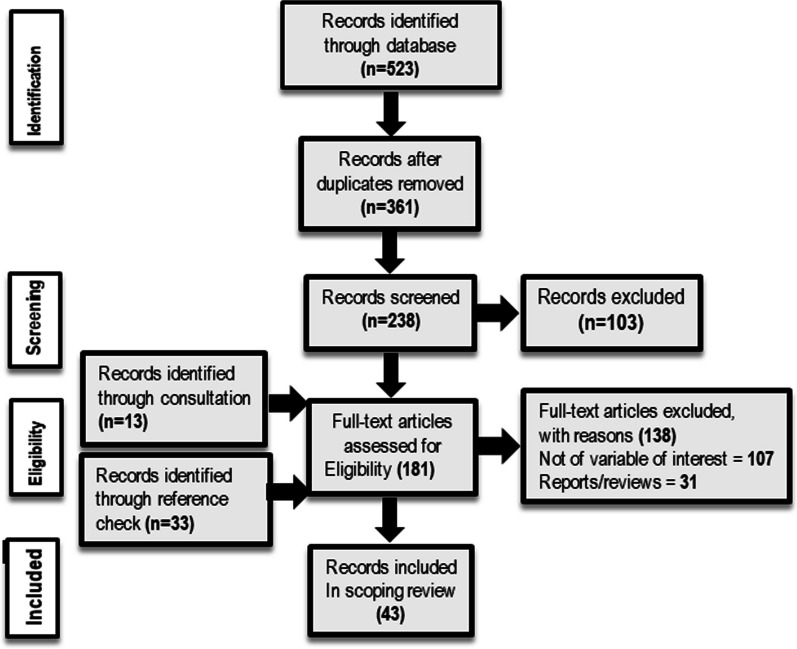
PRISMA flow diagram

This paper was also guided by the Preferred Reporting Items for Systematic Reviews and Meta-Analyses extension (PRISMA) [22, 23]. We sourced peer-reviewed records from the following databases/search engines/publishers: PubMed, Cochrane Library, ScienceDirect, Dimensions, Taylor and Francis, Chicago Journals, Emerald Insight, JSTOR and Google Scholar (see Fig. 1 and Table 1). To guarantee rigidity and comprehension in the search procedure, medical subject heading (MeSH) terminologies were used. The search was conducted at two levels: level one applied the terms “Health policy*” OR “Health politics*” OR “Policymaking*” OR “Policy-making*”, which produced 186 articles. At the second level, additional MeSH terms were introduced: “Africa” OR “Developing countries” OR “Health Care reforms/organisation & administration” OR “Health care sector/standards” OR “Leadership” OR “Evidence-Based Medicine” OR “Public Health” OR “Public policy” OR “Sub-Saharan Africa” OR “Health promotion/trends”, across the databases/search engines/publishers which also yielded 523 articles (see Fig. 1 and Table 1). The scope of the search spanned articles published between 1 January 2010 and 31 December 2023, with searches carried out between 1 November 2022 and 31 January 2024.

**Table 1:** Search strategy

Search strategy item	Search strategy
Databases/publishers/ Search engines	PubMed: 86 peer reviewed articles; Cochrane Library: 23; ScienceDirect: 29; Dimensions: 34; Taylor and Francis: 72; Chicago Journals: 17; Emerald Insight: 34; JSTOR: 95 and Google Scholar: 133
Language filter	English
Time filter	1 January 2010–31 December 2023
Spatial filter	Africa
MeSH terms used	“Health policy*” OR “Health politics*” OR “Africa developing countries” OR “Policy making*” OR “Health Care reforms/organisation & administration” OR “Health care sector/standards” OR “Leadership” OR “Evidence-Based Medicine” OR “Public Health” OR “Public policy” OR “Sub-Saharan Africa” OR “Health promotion/ trends”
Inclusion criteria	(1) Peer-reviewed articles (PRAs) on Africa; (2) PRAs on politics–evidence conflict in national health policy-making and written in the English Language, covering 1 January 2010–31 December 2023; (3) PRAs must provide details on the author(s), purpose/aim, methods/setting, nature of politics–evidence conflict, type of health policy, consequences of politics–evidence conflict and conclusion/recommendation
Exclusion criteria	(1) Media reports; (2) grey literature; (3) PRAs published before 1 January 2010 and after 31 December 2023; (4) PRAs outside of Africa; (5) articles devoid of details on author(s), purpose/aim, methods/setting, nature of politics-evidence conflict, type of health policy, consequences of politics–evidence conflict and conclusion/recommendation; (6) reviewed articles and (7) articles with a low-quality rating

Following the initial search, duplicate articles were imported into and merged in the Mendeley. To attain rigour in the screening, four authors (2, 3, 4 and 5) did the initial screening of titles and abstracts. Subsequently, all articles that passed the inclusion criteria (defined below) were thoroughly reviewed. Doubts over the qualification of an article were resolved by two authors (1 and 6) through detailed discussion until a consensus was reached. With the PRISMA protocol, citation chaining was conducted on all papers that qualified, to identify additional useful articles for further assessment. The first author led, supervised and substantially helped to resolve all discrepancies during data extraction and quality assessment processes.

### Inclusion criteria

Studies about Africa that explored evidence–politics conflict in national health policy-making processes and written in the English language only were included for studies done between 1 January 2010 and 31 December 2023. Additionally, articles must have given details on the author(s), purpose/aim, methods/setting, nature of politics–evidence conflict, type of health policy, impact of politics–evidence conflict and conclusion/recommendations.

### Exclusion criteria

We excluded articles that were not peer reviewed and those with substantial limitations or poor quality. Also, commentaries, grey literature, opinion pieces and media reports were excluded from this review. Articles on politics–evidence conflict that were not conducted in Africa were excluded.

### Quality rating and assessment

Using the procedure of Tricco et al. [22], articles that met the inclusion criteria went through quality rating. Articles that provided background, aims/objectives, context, appropriateness of design, sampling, data collection and analysis, reflectivity, the value of research and ethics were accepted. Thus, the articles were judged and total scores assigned based on the majority of the sections. Articles that scored “A” had few or no limitations, “B” had some limitations, “C” had significant limitations but possessed some relevance and “D” contained substantial flaws that could undermine the findings of the study. Therefore, articles that scored “D” were excluded from the review.

### Data extraction and thematic analysis

Five authors (2, 3, 4, 5 and 6) independently extracted the data. Three authors (2, 3 and 4) extracted data on the author(s), purpose/aim, methods/setting, nature of politics–evidence conflict and type of health policy. Meanwhile, two authors (5 and 6) extracted data on the impact of politics–evidence conflict and conclusions/recommendations (see Table 2). Data extraction was done under the supervision of authors 1 and 6. Using the method of Braun and Clarke [23], a thematic analysis was conducted by three authors (1, 4 and 6). Therefore, data were coded and the themes emerged inductively and yet guided by the stated research questions. The data analysis commenced with multiple readings of the text to familiarize with the data, we then created initial codes, critically assessed the themes, revised the themes, defined and labelled the themes, and produced the report. Additionally, the emerged themes went through exhaustive discussion by all authors to reach a consensus. The themes further went through repeated reviews based on new data until the final themes emerged.

## Findings

The study explored the extent and type of evidence that exists on the conflict between politics and scientific evidence in the national health policy-making processes in Africa. We included 43 papers published between 2013 and 2023. Out of the total, 11(25.6%) [24–34] originated from Western Africa only, 11(25.6%) [35–45] from Southern Africa only, 11(25.6%) [46–56] from Eastern Africa only and 3(7.1%) [57–59] from Eastern and Southern Africa. Also, 1(2.3%) [60] from Northern only, 1(2.3%) [61] from Central only, 1(2.3%) [62] from Eastern and Western, 1(2.3%) [63] from Western and Northern, 1(2.3%) [64] from Western and Southern, 1(2.3%) [65] from Central, Eastern, Western and Southern, and 1(2.3%) [66], thus, cutting across the entire continent. The approaches adopted by these articles included quantitative 2(4.5%) [39, 66], qualitative 39(91%) [24–32, 35–38, 40–51, 53–65] and mixed method 2(4.5%) [33, 52]. The findings are presented by the nature of the conflict, drivers, barriers and impacts of politics–evidence conflicts on national health policy-making in Africa.

### Nature of politics–evidence conflict in national health policy-making in Africa

Our review revealed some form of competing interest in national policy dialogue, from formulation to implementation phases. The most commonly reported conflicting issues include confusion about policy priorities with political ideologies, donor interests and influences [24–30, 51]. We also found that moral, legal restrictions and government priorities are key competing interests in most African countries concerning national health policy-making. Wanjohi et al. [28] noted that governments have competing roles, for example, the sugar-sweetened taxation policy in Kenya.

We found a persistent poor government commitment in most public health policy initiations, formulations and implementations [24, 26, 29, 31–38]. Again, most public policies were formulated and implemented based on the motivation of policy-makers’ financial incentives as well as government and foreign policy conditionalities [27, 30, 36, 37, 39].

It emerged also that donor financing facilitated the process of national public health policy, but strategically skewed the power balance and goals of some health policies [24, 40, 41]. In some cases, donor powers were used in the positive direction, but negatively in others [29, 41–44]. Moreover, power imbalances associated with funding sometimes disrupt the flow of funds from donors, something which negatively affects the content and original purpose of national public health policies in Africa [41, 42, 45].

### Drivers of public health policy-making in Africa

We found that political commitment and strong collaboration among actors, stakeholders and development partners drive public health policy formulation [29, 33, 34, 36–38, 44, 46, 62]. Also, strong existing policies and legal frameworks, employing evidence-based approaches in the policy formulation process, self-sufficiency, prioritization of basic needs and acknowledging weaknesses in the national system and taking measures to resolve such improve policy dialogue [29, 34, 44].

It also emerged that strong government leadership and empirical-based approaches, effective engagement of experienced stakeholders, policy alignment with political priorities and evidence-based interventions are effective in policy formulation [46]. Furthermore, social, political, economic and institutional factors influence the effectiveness of national policy processes [35, 39, 47, 49, 50]. Additionally, the integration of national policies into international ones and formulation of new policies were driven by stakeholder interests, advocacy and collaborative efforts from civil society and the global advocacy movements [34, 47, 66].

### Barriers to national public health policy-making in Africa

We found that fragmented stakeholder interest, institutional responsibility and accountability, inadequate understanding and interpretation of context by stakeholders divided perspectives of actors on the policy context, and lack of understanding among actors about how policy should be financed limits national public health policy formulation [27, 30, 41, 51–53]. Moreover, incomplete and inaccurate data, poor management of resources, improper coordination and communication between actors, inadequate consultation with relevant stakeholders and inconsistency among actors during policy dialogue and formulation processes are also critical limitations to policy formulation [26, 40, 41, 52–55].

Furthermore, it emerged that a lack of strategic leadership and a clear action plan regarding policy processes, poor characteristics of political players and difficulty in understanding and interpreting context by stakeholders frustrate policy processes [25, 28, 32, 35, 37, 53, 54, 59]. Additionally, lack of decentralization of policy formulation and implementation, poor resource mobilization and lack of political engagement with policy beneficiaries, implementers and other relevant stakeholders suffocate some policies in the continent [38, 51, 53, 56]. Besides, most national public health policy processes were motivated by policy-makers’ financial incentives as well as government and foreign policy conditionalities in Africa [27, 36, 40, 46, 57].

### Impacts of politics–evidence conflict in public health policy-making

There are delays in policy dialogue and implementation due to disparities between policy expectations and actual practice, weak policy processes that do not take into account the relevant actors and lapses in policy context [24, 42, 48, 51, 55]. We also noticed delays in the policy formulation process due to high political and external conflict [25, 28, 30, 37, 53]. Moreover, due to poor engagement with policy players, fragmented governance and weak monitoring systems, some health policies do not meet their intended purposes [33, 37, 38, 41, 43–45, 54, 57–59].

Fortunately, evidence suggests a consensus among actors in the policy process which promotes collective ownership, high political commitment, pressure from civil society and other relevant stakeholders as well as policy alignment with political priorities that led to some successes in public health policy formulation [29, 31–34, 46, 51, 60].

## Discussion

Strong empirical evidence is the backbone for impactful national health policy. Pursuant to this, we explored the extent of evidence of conflict of politics and scientific evidence in the national health policy-making process in Africa. The study revealed four significant themes under which the discussion is organized. These include political influence in public health policy, drivers of public health policy, barriers to effective public health policy and impact of politics–evidence conflict on public health policy-making process in Africa.

### Political influences in public health policy

We report that public health policies in Africa are heavily influenced by politics rather than scientific evidence. Most governments succumbed to donor interest which largely define the policy process [28, 30, 37]. This is consistent with several previous studies [1, 13, 19, 20] who reported similar findings. Meanwhile, the interests of these donors are often at variance with the health needs of the citizenry in Africa. Moreover, affirming previous studies [13, 19], our current study exposed the influence willed by donors which reiterates the power imbalance and lack of independence that sometimes characterizes policy-making in Africa to the detriment of its local needs.

In contrast to the findings of the current study, an earlier study [20] recognized the role of political ideology in the national health policy process. Meanwhile, the reviewed articles [24, 45, 47, 48, 57] showed that political influence undermines policy formulation that cater for sustainable health development in the continent. For instance, Oleribe and colleagues [67] attributed the failure of abortion policies in Burkina Faso and other sub-Saharan African countries to political influence and poor stakeholder consultation. This could be largely due to limited financial capacities and impositions by global organizations and allegiances.

### Drivers of public health policy

We found that, generally, the main drivers of public health policies in Africa include health organizations, donor agencies, development partners, political manifestos and nongovernmental agencies [34, 35, 38]. For instance, a review of the National Health Insurance Policy of Ghana revealed that its success stemmed from its alignment with the political manifestos of successive governments [36]. This agrees with previous studies [2, 3] which revealed how donor agencies, development partners and political manifestos drove public policies. Furthermore, global agencies such as WHO, United Nations Development Programme (UNDP) and others collaborate very much to drive the health policy directions, especially the less developed nations, in meeting global health standards such as the SDGs for health. Thus, strong advocacy and partnership influence policy direction in all affiliate countries.

### Barriers to effective public health policy

The main barriers affecting an effective public health policy-making process in Africa include poor consultation, orientation and decentralization [26, 29, 36]. Typically, political policies are expected to promote stakeholder consultation, orientation and proper decentralization of health interventions. However, we found that political policies and activities are rather undermining stakeholder consultation, orientation and proper decentralization of health interventions [26, 29, 36]. This strongly upholds findings from previous studies [3, 5, 15, 16] that political interests undermined successful implementation of national policies. This is mostly because governments in Africa lacked adequate resources and empirical evidence to effectively drive health policy process to a successful implementation [30, 41, 45, 47]. Unfortunately, disparities in expectations and systems account for lapses in the policy context and the entire formulation process [28, 32, 44, 53, 61, 62]. Some previous studies [5, 16] have reported how implementation of public policies fell short of public expectations. Moreover, leadership corruption, inadequate consultation, poor advocacy and inadequate use of core evidence in policy-making and implementation compromise the future of public health in Africa.

### Impacts of politics–evidence conflict on public health policy

We found that competing interests, political dishonesty and lack of political commitment affect the expected health needs of many African populations [24, 26, 51]. Poor coordination of key policy actors such as donors and other stakeholders, coupled with poor integration of global goals into local health policy frameworks undermined intersector participation which results in poor health policy formulation and implementation [24, 26, 51]. According to previous studies [15, 68], health policies of most developing countries are driven by political propaganda that often does not deal with the critical health needs of the citizenry. Meanwhile, the evidence is that in a few countries where health policies are led by empirical evidence and supported by political commitment, health indicators improved [63, 64]. For instance, we found that in Kenya, Ethiopia and South Africa, effective participation of healthcare professionals and other key stakeholders in developing policies on abortion lead to significant reductions in mortality due to illegal abortions [63, 64]. Then, effective stakeholder participation becomes key to the success of public policy implementation [15]. Therefore, effective decentralization and stakeholder participation, specifically the targeted beneficiaries and healthcare professionals, are necessary to engender effective policy implementation.

### Alignment of findings with policy frameworks

The current review aligns well with Walt and Gilson’s policy framework. First, we found that donor interests, mostly championed by political interests (policy context), largely define most national health policies in Africa [28, 30, 37]. Second, rather than evidence-based policy objectives and legislations, the contents of most national health policies in Africa do not align well with the needs of the locals (policy content). This creates misalignment in policy in objectives and content to the detriment of the health of the African citizen. Thus, the content of most national health policies failed to address the critical health needs of the ordinary African citizen. Third, such policies are driven by donor interests that are supported by political interest (policy process) into key policy activities from policy conception, formulation, negotiation and communication to evaluation [24, 26, 51]. Fourth, on the policy actors, key stakeholders such as health professionals, academia and community leaders do not actively participate in the policy process in Africa. This ultimately undermines policy ownership and implementation success [5, 16].

## Strengths and limitations

This study is a significant addition to existing empirical accounts of the politics–scientific evidence conflict in the national health policy-making process in Africa. To uphold compression and rigour in the search procedure, we applied the MeSH terms in search of only peer-reviewed articles on the variables. Additionally, we set inclusion and exclusion criteria, and the reviewed articles went through a rigour of quality rating, using standardized guidelines. Moreover, data extraction was independently conducted and verified by all authors. These notwithstanding, there are limitations worth acknowledging. First, including only peer-reviewed articles that are written in the English language and covering only Africa may have limited the literature samples used in this study. Thus, some excluded articles, written in other languages, may contain important details. Moreover, we acknowledge that the inherent weaknesses and biases in the reviewed articles are carried into our research.

## Recommendations for policy direction and research

Based on the findings from this review, we reiterate evidence-based agenda setting before any policy process. More importantly, policy-makers should research and establish strong evidence to demonstrate the viability of the proposed policy. Additionally, government and political leadership may want to limit corruption and be committed to ensuring that the policy agenda meets the immediate local needs and that global policy initiatives do not undermine pressing domestic health needs. Furthermore, it is recommended that all stakeholders, including implementers and beneficiaries, are engaged and fully participate in the entire policy process. Finally, we recommend that all stakeholders have a common understanding of the policy agenda and what is expected to achieve, and remain resolute to the collective purpose through the policy processes.

## Conclusions

The extent of political–evidence conflict in national health policy processes is marked by obstructions. There are issues related to corruption, where political interests prioritize their financial gain over the needs of the healthcare system and the public. The potential impact on health in Africa is an increase in disease burden, lack of productivity and lack of progressive health development. There is largely inadequate funding for healthcare, a situation that is resulting in poor public health policy implementation leading to inadequate availability of essential medicines and supplies, and causing other negative impacts on public health.

Addressing these conflicts between political interests and evidence into health policy formulation and implementation require strong leadership, effective governance and commitment to the public health agenda. It also requires collaboration between and among different stakeholders, including government officials, healthcare providers, researchers and civil society organizations. By working together, it is possible to develop policies and strategies that are evidence based, equitable and sustainable, that promote the health and well-being of all persons in Africa.

## Data Availability

All relevant data provided in the appendix.

## Appendix

See Table 2.

**Table 2:** Extracted data

Author/year	Purpose/aim	Methods and setting	Nature of conflict	Policy type(s)	Consequences of conflict on policy	Conclusion
(Abubakar et al., 2021)	Assess the pros and cons of evidence generation and decision-making, discuss potential impacts and derive lessons for Nigeria, sub-Saharan Africa, and beyond	Qualitative/Nigeria	Challenges with the coproduction model included lack of transparency, bureaucratic barriers and a narrow focus on the direct health impacts of the disease rather than considering the broader social and economic effects of response measures	COVID-19 response guidelines	The guidelines helped in achieving its goal of slowing down the spread of the virus However, COVID-19 has inflicted significant social and economic hardships on many Nigerian households and individuals, complicating efforts to address the country’s broader health challenges amidst economic contraction	A multidisciplinary approach, integrating epidemiological, social science and economic analyses, along with evidence coproduction with policy-makers, is key to addressing the dual social and public health challenges of the COVID-19 pandemic in Nigeria and beyond
(Agyepong et al., 2021)	To explore why and how collaborations and fragmentations occur during national policy and programme agenda setting	Qualitative/Ghana and Sierra Leone	1.Country and global level factors 2.Power imbalances attached to funding 3.Disruptions in the flow of funds 4. Lack of political commitment and prioritization	Health Policies UHC HS HP	Accountability and trust issues collapse the synergy in the entire process of policy-making	Synergies and fragmentations are prevalent in policy agenda setting, formulation and implementation Fragmentations are detrimental to achieving policy goals Country actors require commitment, technical expertise and leadership to tilt the power balance towards Prioritization and political commitment of country actors
(Alhelou et al., 2022)	Explore the opportunities and challenges countries have experienced and identify gaps to advance policy-making on menstrual hygiene and health	Qualitative/India, Kenya, Senegal and the United States	Policy drivers identified include: 1.Strong leadership and political will 2. Employing evidence-based approaches 3. Non-dependency/self-sufficiency 4. Prioritization of basic needs 5. Acknowledging weaknesses in the system and resolving them	Menstrual hygiene and health policies	Policies are being implemented smoothly but with some systemic challenges	African countries can initiate, formulate and implement policies to solve complex health problems with political will and minimal external interference
(Akhnif et al., 2020)	Document the developmental process of a consolidated health financing strategy in Morocco	Qualitative/ Morocco	Success factors: 1.High political commitment 2. All actors adopted a common path of action	Health financing policy	Collaborative nature of the process favoured consensus building and collective ownership of the policy	The policy document was produced through collaboration among actors The process was devoid of the usual top-down approach and was more participatory in nature
(Amukugo et al., 2021)	Analyse Namibia’s policies on NCD prevention Assess the government’s readiness to adopt a sugar-sweetened beverage tax policy	Qualitative/Namibia	The lack of interest on the part of government	No progress towards the enactment of the sugar-sweetened beverage policy		Although there is the endorsement of the sugar-sweetened beverage (SSB) policy, there are no steps to its adoption due to lack of convincing data on its impact This makes advocacy difficult and policy actors are latent
(Blystad et al., 2019)	Examined the relationship between the abortion law, policy and access to safe abortion services	Qualitative/Zambia, Tanzania and Ethiopia	Ambiguity of the language used in the laws and policies Complexities between the laws, policy and actual access to health services	Abortion law Safe Abortion policy		
(Colvin et al., 2021)	Explore the barriers to and opportunities for improvement of, effective TB infection prevention and control	Qualitative/South Africa	Barriers include fragmentation of institutional responsibility and accountability for TB-IPC Inability of advocates to present TB-IPC as an urgent policy problem Barriers to policy innovation from lack of evidence to justify new policy	TB Infection Prevention and Control		TB-IPC is a chronic and complex health system challenge and requires both policy- and behavioural-level interventions. There is the need to effectively deal with the upstream barriers to policy formulation and implementation
(Croke, 2020)	Identified the success factors of model primary healthcare programmes in Ethiopia	Qualitative/Ethiopia	Political will, Characteristics of political leaders A clear strategic plan of action informed by several policy experiments over a period	Primary healthcare programme	Tremendous success of the model programme informing its replication at the national level	Government models of federalism and policy to involve the population in service delivery models, to ensure political stability and development yielded the desired results
(Dalglish et al., 2017)	Examine processes of health policy development in a case study of Niger	Qualitative/Niger	Government policy-makers showed adeptness in negotiating with donors and weighing conflicting factors but lacked the capacity and resources to formally evaluate and document programmes, limiting their ability to draw reliable lessons from them	Community case management of childhood illness (iCCM) policy	Government actors employed logical and ethical reasoning in making decisions that were subsequently identified as crucial to the success of iCCM	Access to codified knowledge empowers participants in policy discussions, yet it constitutes only one facet of knowledge utilized in the policy process, potentially not the most significant
(Ditlopo et al., 2013)	Examine and identify the implementation and success factors of financial incentives among nurses	Qualitative case study/South Africa	Weaknesses identified include incomplete and inaccurate data on specialized nurses. Poor management of time and resources, improper coordination and communication. Rush in implementation, lack of consultation with health facility managers and poor orientation for implementors	Occupation-specific dispensation (OSD) policy; a financial incentive policy for health works in the public sector	Suboptimal implementation of the policy	Successful implementation of financial incentives requires adequate planning and management to maintain the morale of staff and reduce their grievances
(Ditlopo et al., 2014)	Analyse the dynamics, strengths and weaknesses of nurses to participate national health workforce policies	Qualitative/South Africa	Poor awareness creation among policy beneficiaries Lack of consensus among policy actors No representation of the policy beneficiaries in policy-making process Lack of consensus over which nursing group represents nurses in policy-making	Policy regarding nursing practice, scope of practice framework for nursing qualifications and renumeration policy	Negative effect on how nurse’s views and inputs were regarded and included into the policy	There is the need for strong leadership, improved health policy capacity and skills of nurses to contribute effectively to health policy
(Etiaba et al., 2023)	The study analyses government collaboration in implementing maternal, neonatal and child health (MNCH) programmes, derived from an integrated strategy. It aims to identify principles applicable to multilevel governance, especially in low-income countries	Qualitative case study/ Nigeria	Memoranda of Understanding were signed collaboratively but remained unimplemented. State did not meet programme goals due to a disconnect in the national governance structure, despite contextual differences	Maternal, neonatal and child health (MNCH) policies	Misaligned governance structures constrained implementation	Resource-limited countries need ongoing advocacy and tailored models for distributed leadership across government levels. Stakeholders must understand available collaboration drivers and system context requirements
(Gavriilidis & Östergren, 2012)	Analyse the ATM policy	Quantitative/South Africa	Facilitators: importance of the policy to communities, employment, education promotion, entrepreneurship and resource mobilization at the periphery Barriers: centralised conception, planning and implementation, lack of local adaptations, authoritative legislation	African Traditional Medicine Policy		There is the need for deliberate community representation in policy-making, through conception, design to implementation. This participatory approach improves
(Haaland et al., 2020)	Address gaps between knowledge, policy and practice Explore processes involved in translating policy into practice	Qualitative/Zambia	Issues of morality are key barriers to the policy implementation	Regulate and ensure safe abortion services	Healthcare facilities do not make safe abortion services available to women who seek them especially in rural areas	The discourse on safe abortion is even during policy meetings due to the dominant moral regime in Zambia
(Holcombe & Gebru, 2022)	Describe the actors and processes involved in the policy process	Qualitative/Ethiopia	Drivers of the process: Government’s receptiveness, the pressure from civil society, health professionals and other NGOs	Safe abortion policy to promote the access to safe legal abortion services to prevent maternal mortality	Policy process was smooth and fast	The policy was internally driven with minimal external influence. There was a strong collaboration between government and civil society, and this is the major driver of the success achieved
(Hussein et al., 2021)	Describe how the development of a community health policy contributes to attaining UHC and PHC targets in Kenya	Qualitative/Kenya	Drivers: strong government leadership, strong partnership, strong stakeholder engagements Alignment with political priorities Decentralization of the context to identify specific community needs The policy development was guided by research and evidence	Community health strategic plan National health sector strategic plan,	Successful policy development	The various contributing factors to the successful policy development are useful lessons for other similar jurisdictions. However, the successful implementation of the policy will depend the sustained political interest and consistency in financing sources
(Jacobs & George, 2022)	Youth participation in the formulation of the Adolescent and Youth Health Policy was examined	Qualitative/South Africa	Barriers: donor politics and segmented donor priorities, fragmented among actors on how to embrace diversity and difference and how to handle power relations Drivers: opening of a good policy window, consensus between policy actors, youth participation	Adolescent and Youth Health Policy		The opportunity for youth to participate in the policy development was a great achievement. However, there is the need to ensure proper representation of the youth in all the concerns them. This is a fundamental human right
(Kagaha & Manderson, 2021)	Explored the role of power operations in setting priorities for maternal healthcare	Qualitative (ethnography and discourse analysis)/Uganda	Moral and legal restrictions Government priorities	Abortion care policy		
(Kielmann et al., 2021)	Assess health systems readiness to implement a new policy in resource constrained settings	Qualitative/South Africa	Facilitators: interventions to address staffing issues were addressed, infrastructure redesign and equipment arrangements	Policy on drug-resistant TB management	The programme was implemented successfully	Healthcare workers were willing to deliberately go the extra mile to do things that would ease the implementation of the policy
(Koduah et al., 2018)	Understand decision-making processes that influence policy agenda	Qualitative/Ghana	Understanding and agreeing on the context, stakeholder interactions, ideas and framing of issues	Maternal health policies	Effective use of power to convince final decision makers moves the decision into a specified direction	Interconnectedness of policy drivers is such as the context, actors and their powers crucial in the policy process
(Koon et al., 2020)	Providing social explanation to the president’s decision not to sign a policy	Qualitative/Kenya	Actors’ preferences influenced their judgement. Decisions were not evidence informed but were informed by the values of the actors. There was no understanding of how to finance the policy among actors	National Social Health Insurance law	The president refused to sign the document into law	The Ngilu Bill did not fail, but rather was fragmented into several smaller policy positions, some of which have recently been legislated
(Kumwenda et al., 2021)	Explored factors that influenced development, adoption and implementation of ART policies	Qualitative/Malawi, Tanzania and South Africa	Weak health systems, suboptimal care Pressures from different stakeholders to accelerate or slow implementation Donor financing facilitated the process but skewed the power balance	Anti-retroviral therapy policies	Donor influence interfered with local innovative solutions to address specific local health system issues	Donors should be more focused on comprehensive health systems strengthening to achieve more effective results
(Mac-Seing et al., 2022)	Explored policy actors’ perceptions of pro-disability laws, barriers to SRH services for people with disabilities and recommendations for addressing inequities	Qualitative/Uganda	Legislation and policy implementation faced technical and financial challenges, with disability issues lacking prioritization. People with disabilities encountered various barriers accessing SRH services, including physical, attitudinal, communication, and structural obstacles	Sexual and reproductive health (SRH) policy	There is proof of the complex challenges individuals with disabilities encounter when accessing SRH services, alongside the struggles in implementing disability-focused policies in Uganda	Policy actors identified and proposed concrete solutions to mitigate health inequities for people with disabilities
(Masefield et al., 2021)	Explored how local stakeholders perceive their involvement in shaping the National Health Policy II (NHP II) and Health Sector Strategic Plan II (HSSP II)	Qualitative/Malawi	Tokenistic involvement, stakeholder hierarchy, mutual distrust, preferred stakeholders, no culture of engagement	National Health Plan II (NHP II) and Health Sector Strategic Plan II (HSSP II)	After18 months of policy implementation, stakeholders observe minimal improvement in governance and lack confidence in the government’s ability to achieve Universal Health Coverage (UHC)	Stakeholders expressed that both top-down and bottom-up pressures were lacking, resulting in superficial consultations with local stakeholders. As a consequence, these stakeholders felt powerless to influence health policy-making in a manner that aligns more effectively with the needs of their respective communities More inclusive top-down efforts are needed for effective stakeholder engagement
(Mauti et al., 2022)	Assess how the adoption of the HiAP approach can leverage on SDGs implementation in Kenya	Mixed method/Kenya	Strong political commitment Draw on existing structures Funding strategy was not captured the framework	Health in all Policies		Multisectoral approach is a prerequisite for a successful comprehensive policy. Fragmentation of the various sectors will be a disadvantage to policy development and implementation
(Modisenyane et al., 2017)	Understand how South Africa integrates domestic health policy into its foreign policy	Qualitative/South Africa	Social, political, economic and institutional factors influence the integration process The integration process was driven by national and external developments, stakeholder interests and advocacy and collaborative efforts from civil society as well as global advocacy movement	Antiretroviral Policy	Global pressures to make access to antiretroviral treatment a fundamental human right compelled the local health system to prioritize the ARV policy	Global and transnational systems can influence local policy focus by changing policy priorities
(Mukuru et al., 2021)	Examine the roles of policy elites and how their interests drove maternal health policies	Qualitative/Uganda	The policies were driven by senior MoH officials, cabinet members, health development partners	Maternal Health Policies	The policies were skewed towards elite personal political and economic interests rather than reducing maternal mortality	The policies on maternal health in Uganda were informed by the personal interest of elites and not by the aim of reducing maternal mortality
(Murray & Rutland, 2022)	Tests hypotheses from public health policy-making literature concerning the influence of medical, political, social, economic and external factors on African countries’ issuance of Stay-at-Home Orders (SAHOs) in response to the early COVID-19 pandemic	Quantitative/54 African countries	The analysis indicates that medical factors and external influences significantly shaped decisions, whereas political factors had minimal impact. Social and economic factors did not appear to play a significant role	Stay-at-Home Orders (SAHOs) in response to the COVID-19 pandemic	While effective in curbing disease transmission, these measures have considerably disrupted social and economic structures	African leaders balanced competing factors during the initial phases of a public health crisis
(Mwisongo et al., 2016)	Understand the how power influences policy processes	Qualitative/Cabo Verde, Chad, Guinea, Liberia and Togo	Different types of power were used Positive or negative power, transitive, disproportional and structural power	National policies	When negatively applied, can influence and slow down the entire process as well as change its focus	Power could positively or negatively influence the policy process, at various stages of the process
(Novignon et al., 2021)	Document the political path to the establishment of the Ghana National Health Insurance Scheme (NHIS)	Qualitative/Ghana	Drivers: an open policy window Government’s political will (manifesto promise) Strong collaboration between the actors on financing the policy Massive interest and support from development partners Politics cut across the entire policy process External actor politics, beneficiary politics, interest groups, leadership politics, budget politics, bureaucratic politics	NHIS	The strong politic interest of the sitting government and the opposition facilitated the development of the policy without unnecessary delays	Ghana’s NHIS policy development and implementation benefited from a good political atmosphere and leadership drive to achieve a manifesto promise. In addition, the engagement and collaboration between the internal and external actors was crucial to its success. Finally, the willingness of stakeholders to manage fragmentations to arrive at compromises easily facilitated the process
(Okedo-Alex et al., 2021)	Assessment of the status of domestic funding and advocacy Strategies for improving funding of Health Policy and Systems Research (HPSR)	Qualitative/Nigeria	Barriers of domestic funding of HPSR are political and policy transition, corruption and bureaucratic bottlenecks	Health policies	Lack of domestic funding of HPSR is affecting the generation of strong evidence base data to inform and guide policy	Improved private sector involvement, continuous advocacy, multi-stakeholder coalitions for advocacy and researcher skill building on advocacy were suggested as the way forwards
(Oraro et al., 2020)	Examined actor values and interest that influence agenda setting in health financing	Qualitative/Kenya	Lack of strategic leadership Fragmentation of stakeholders Understanding and interpretation of context by stakeholders	Universal Healthcare Policy	Conflicts in priorities of key actors threatens smooth progress towards the expansion of healthcare coverage as well us financing it	Properly contextualizing the competing health needs and prioritizing them in the policy arena is a more practical way of achieving results
(Parkhurst et al., 2021)	Explore the impact of national and global network and related politics on malaria control in sub-Saharan Africa	Qualitative/sub-Saharan Africa	Balancing global goals with local realities Managing expectations and coordination of non-state stakeholders Peculiar NMCP institutionalized systems, structures and processes capable of influencing local capacity building			Although NMCPs are very well structured and adequately resourced by global partners and Ministries of Health, the programme can not be isolated from the local contexual realities
(Ridde & Faye, 2022)	Understand how the COVID-19 response policy was formulated in Senegal	Qualitative/Senegal	Leadership and coordination conflicts Inadequate community participation and engagement with scientific community Power dominance by international donor	COVID-19 policy	The highly centralized and biomedical processes employed in the development of this policy, with very little intersectoral involvement and contribution from the scientific community, resulted in challenges with implementation	Policy-making should be guided by evidence and context, not only politics
(Ruhara et al., 2021)	Described the policy landscape, identify and analyse the facilitators of and barriers to strengthening taxation on sugar-sweetened beverages	Qualitative/Rwanda	Government commitment to boosting sugar production Failure of existing policy to identify sugar as a risk factor for noncommunicable diseases	Tax to reduce the consumption of sugary beverages		The setting for sugar-sweetened beverage tax strengthening is a very complex There are several impediments as well as facilitators in the policy environment to strengthen the existing tax
(Sambala & Manderson, 2017)	Examine the public health policy perspectives on vaccination to prevent the spread of infection under post-pandemic conditions	Qualitative/Ghana and Malawi	The programme was motivated by policy-makers own financial incentives as well as government and foreign policy conditionalities	Policy on influenza virus vaccination	Confusion about the targets and the coverage among the policy actors. For some policy-makers the process was successful, but others think otherwise	The vaccination intervention was problematic, its implementation was too late
(Omar et al., 2010)	This paper delves into the formulation of suitable mental health policies and their successful execution	Qualitative/ Ghana, South Africa, Uganda and Zambia	Mental health policy processes in all four countries were inadequate, leading to either weak or non-existent policies, with an impact on mental health services	Mental health policy	Health policy processes frequently lack strength and adequate resources, especially in the realm of mental health. This deficiency often leads to bureaucratic delays and under-utilization of available evidence	More work is needed to improve mental health policy processes in African countries. Involving a wider range of stakeholders is crucial due to the diverse nature of mental health issues
(Seddoh & Akor, 2012)	Conceptualize the various levers of policy formulation	Qualitative/Ghana	Various stakeholders, that is technical expects, civil society, academicians and politicians, had their various interests	NHIS		During the policy process, it is important that the actors consider the content as well as the context to appreciate the viewpoints of others
(Shiroya et al., 2019)	Presents Kenya’s experience of translating the UN declaration to national policies for diabetes prevention and control	Qualitative/Kenya	Open policy windows, political drive triggered by diabetes community, Scant local evidence	Policy for noncommunicable diseases	Suboptimal gains due to poor engagement with and participation of non-healthy sector players, fragmented health governance and week monitoring systems	Contrary to global recommendations, the policy process was largely driven by the health sector without the involvement of other sectors. Efforts to achieve population-wide impart will be enhanced when other sector players are brought on board
(Simen-Kapeu et al., 2021)	Examined the process of developing community health policies to extract insights from Liberia’s efforts to strengthen its health system after the Ebola Virus Disease (EVD) crisis	Mixed method/Liberia	Establishing a coordination mechanism and harnessing partnership support, adopting a systemic approach to better guide policy changes, enhancing community involvement and conducting planning based on evidence to advise policy-makers	Community health policy	The policy was completed and at implementation stage	To enhance resilience against future shocks and bolster primary healthcare (PHC), community-based systems should assume a more significant role. This necessitates viewing communities not merely as recipients of health services but as
(Thow et al., 2021)	Examine how historical economic policy agendas and paradigms have influenced current food and nutrition policy and politics in Ghana	Qualitative/Ghana	Poor integration of nutrition into existing food policies Existing food policies overly focused on food production, creating employment and economic returns at the expense of nutrition sensitive food supply	Food and nutrition policy		
(Wanjohi et al., 2021)	Assess the policy and stakeholder landscape relevant to nutrition related noncommunicable diseases and sugar-sweetened beverage taxation	Qualitative/Kenya	The role of nutrition in noncommunicable disease prevention is not a priority for policy-makers Government has competing roles such as growing the sugar and feed processing industries The dangers of sugar-sweetened beverages have not gained national consensus	Sugar-sweetened beverages tax policy	Taxation of sugar-sweetened beverages is not a policy priority	There is the need for local advocacy in favour of sugary beverage taxation Public and policy-maker education to understand the dangers of sugary beverages and refined foods
(Zulu et al., 2022)	Examined the events, actors, and contexts behind the withdrawal of Zambia’s Community Health Strategy	Qualitative/Zambia	Divided perspectives of actors Numerous international partners with various interests Shifted locus of the strategy at the MoH No service provider and community participation in the policy process	Community Health Strategy	The policy development process was highly political and characterized by fraught external This led to its abrupt termination	Interaction between events, actors and context in policy development cannot be overlooked. The success of these interactions in the driving force behind any successful policy

## Abbreviations

MeSH: Medical subject headings
PRAs: Peer-reviewed articles
PRISMA: Preferred Reporting Items for Systematic Reviews and Meta-Analyses extension
SDGs: Sustainable Development Goals
UNDP: United Nations Development Programme
